# Inhibition of lignification of *Zizania latifolia* with radio frequency treatments during postharvest

**DOI:** 10.1186/s13065-019-0647-y

**Published:** 2020-01-18

**Authors:** Changwen Ye, Chen He, Bowen Zhang, Lixuan Wang, Lufeng Wang

**Affiliations:** 1 Zhengzhou Tobacco Research Institute of China National Tobacco Corporation, Zhengzhou, 450001 Henan China; 2 College of Food Science and Technology, Huazhong Agricultural University, Wuhan, 430070 Hubei China; 3 Key Laboratory of Environment Correlative Dietology, Ministry of Education, Huazhong Agricultural University, Wuhan, 430070 Hubei China

**Keywords:** *Zizania latifolia*, Radio frequency, Lignification

## Abstract

*Zizania latifolia* is easily lignified after harvesting, leading to the degradation of food quality and commercial value. Thus, this study evaluated the effect of radio frequency (RF) treatments on lignification inhibition of *Zizania latifolia*. The results showed that the lignin content of *Zizania latifolia* treated with RF decreased significantly compared with the control group. At the 7th day of storage, the phenylalanine ammonia lyase activity of the 90 W RF treatment group decreased by 52.9% compared with the initial value. The activities of peroxidase and polyphenol oxidase in the stems of *Zizania latifolia* were significantly (*p* < 0.05) decreased after RF treatments. Besides, a decrease in conversion rate of O_2_^−^ and H_2_O_2_ to downstream products was observed, indicating that the related invertases were inhibited by RF treatment. All of these showed that RF treatments contribute to inhibit or delay the lignification of *Zizania latifolia*, providing a better taste and quality for products. 
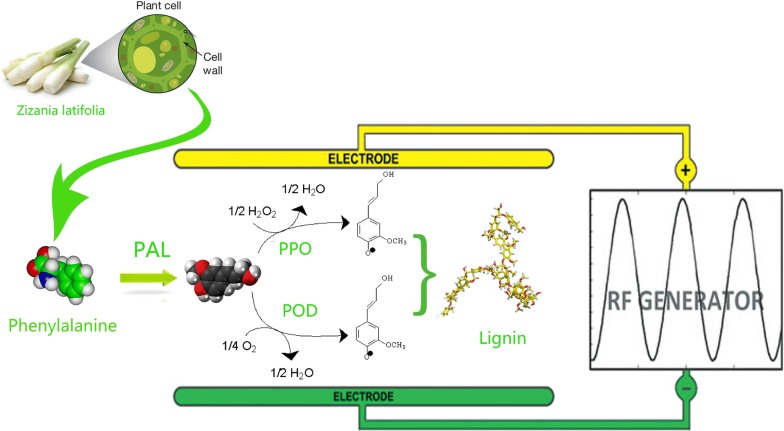

## Introduction

*Zizania latifolia*, also known as Manchurian wild rice or water bamboo shoots, is the only member of the wild rice genus *Zizania* native in Asia. It is widely used as an economical plant, and its stem and grain are both edible. Its edible part is a fleshy stem that grows after being infected by smut [11, 12, 23].

However, *Zizania latifolia* is highly prone to lignification after harvesting with the symptoms of increasing hardness and rough mouth feel, which is attributed to the accumulation of lignin and cellulose in the leukocyte wall [15]. Among them, the biosynthesis of lignin is considered as main reason for lignification [1]. The lignin monomers undergo a series of catalytic polymerization reactions to form a lignin macromolecular polymer deposited on the cell wall, and crucial enzymes involved in the process include phenylalanine ammonia lyase (PAL), peroxidase (POD), polyphenol oxidase (PPO), and acetaldehyde dehydrogenase (CAD) [17]. PAL is the major role for the initiation of lignin metabolism, which catalyzes the conversion of l-phenylalanine to cinnamic acid, and then produces *p*-coumaric acid (a precursor of lignin) [10]. Meanwhile, PPO engages in the reaction of phenolic precursors and the polymerization of lignin monomers [5]. Studies have confirmed that the activities of PAL and PPO gradually increase during postharvest storage [13]. Therefore, the lignification of *Zizania latifolia* may be alleviated in the case where the activities of PAL and PPO are inhibited.

On the other hand, physiological aging caused by free radical damage can also lead to the lignification of *Zizania latifolia*. Active oxygen is an important free radical, mainly including superoxide anion (O_2_^−^) and hydrogen peroxide (H_2_O_2_) produced during the metabolism of fruit and vegetables. Research suggest that the accumulation of active oxygen cause the peroxidation of the lipid membrane, thus resulting in the destruction of cell membrane and accelerated aging [16, 19, 26]. The increase of H_2_O_2_ content will promote ethylene production and accelerate subsequent aging. Liu et al. [13] found that the exogenous H_2_O_2_ treatment could multiply the ferulic acid dimer in plant tissues, and increase the oxidative cross-linking of various phenols and the degree of lignification. Given this situation, active oxygen should be concerned for the lignification process of *Zizania latifolia*.

At present, gibberellin, chlorophyll, 1-MCP and the like have been proven to have positive effects of lignification inhibition, but they introduce foreign chemicals [13]. Research and development of physical methods without the application of foreign chemicals may be potentially valuable to solve the problem of lignification. Radio frequency (RF) heating is a kind of dielectric heating technology. The polar molecules in the material will reciprocate or move when the material is positioned in a high-frequency alternating electric field, leading to the rising temperature due to the collision between molecules [18]. In some ways, RF heating at intermediate frequencies is a more promising technology as its greater penetration compared to microwave heating. In agriculture, RF heating has been widely studied and confirmed as an effective way to kill pests in certain food crops after harvest, such as shelled walnuts [7, 8]. This mainly relies on the fact that even if the material has a large thickness, the RF can easily penetrate.

Although the application of RF has been widely carried out in agriculture, there is limited information about inhibitory effect of RF treatment on lignification of *Zizania latifolia*. At present, it is usually packaged and refrigerated after harvesting to inhibit lignification. However, this method can only partially inhibit lignification and bring higher energy consumption and cost. According to the principle of RF, we speculate that it may inhibit lignification of *Zizania latifolia*. Thus, the aim of this study was to investigate the potential of RF as a protective technology to retard the lignification of *Zizania latifolia,* as well as to explore its effect on the lignin-related metabolic enzymes and reactive oxygen species.

## Materials and methods

### Materials and chemicals

The *Zizania latifolia* were harvested in August 2017 from Fumin Farm (Jiayu, Hubei, China). The plant material was identified as Zizania latifolia, voucher specimen (EJ-2) by Professor Ke Weidong from Wuhan Vegetable Science Research Institute (China) and is now preserved in Herbarium of Wuhan Vegetable Science Research Institute. The shoot were immediately transported to laboratory by car within 3 h and pre-cooled in water at 8–10 °C overnight. Zizania latifolia was selected for uniform shape, color and size (with the diameters 30–35 mm) and the absence of any blemishes or disease. The outer leaf sheaths were carefully peeled off by hand. Then, the raw materials were pre-cooled at 10 °C until the temperature was consistent before RF treatment. Bromoacetyl, *o*-phenylenediamine, α-naphthylamine, β-mercaptoethanol, H_2_O_2_ (30%), polyvinylpyrrolidone (PVP), sulfanilic acid, and ammonia were purchased from Aladdin Biochemical Technology Co., Ltd (Shanghai, China). Phenolphthalein indicator, iron oxalate dehydrate and titanium tetrachloride were supplied by Sigma (Madrid, Spain).

### Radio frequency heating operation

The radio frequency heating equipment (RG-200) was customized and purchased from Zhongshan Ruiyu Electronics Co., Ltd. (Zhongshan, Guangdong, China). It works at 81.36 MHz ± 0.005% with a maximum power of 500 w.

*Zizania latifolia* samples were wetted with tap water and then fully enclosed with 3 mm thick wet cloth. Samples placed between the upper and lower plates of the RF equipment were subjected to 60, 70, 80, and 90 W for different groups with the procedure of 2 min work and 1 min pause (repeated 4 times). After treatments, the samples (include the control group) were transferred into aluminum foil bags individually and storage at 20 °C for 0, 1, 3, 5, 7 days respectively.

### Determination of the lignin content of *Zizania latifolia*

The lignin contents of samples were determined using the method described by Luo et al. [14] with some modifications. About 5 g of samples were extracted 3 times with 50 ml 1% (v/v) 11 M HCl in methanol for 1 h, each time under continuous stirring and centrifuged at 14,000*g* for 10 min. The final residue was used for analysis of lignin. Lignin content was determined gravimetrically after acid hydrolysis of the insoluble-alcohol residue under previously established conditions. This residue was mixed with 12 M H_2_SO_4_ and hydrolyzed for 3 h at 20 °C with stirring. The solution was then diluted with distilled water up to 1 M H_2_SO_4_, and heated for 2.5 h at 100 °C with continuous shaking, cooled, vacuum- filtered through an acid-treated 0.45 μm Millipore HVLP filter, and rinsed with 100 °C distilled water. The filter, containing lignin, was air-dried at 60 °C for 48 h and weighed. Results were expressed as g lignin per 100 g fresh weight.

### Determination of the PAL activity of *Zizania latifolia*

PAL activity was analyzed using the method described by Song et al. [21] with slight modifications. Enzyme extracts were prepared with 2 g of *Zizania latifolia* and 10 mL of 0.1 mol L^−1^ borate buffer (pH 8.8) containing 5 mmol L^−1^ β-thioethanol, 0.5 g polyvinylpyrrolidone (PVP), 1 mmol EDTA. The mixture was homogenized with 2 g of quartz sand in ice bath. Then the homogenate was centrifuged at 6000*g* for 15 min at 4 °C (Allegra X-30R Centrifuge, Beckman, Krefeld, Germany). After filtration using filter paper (D9, Shanghai Sirui Technology Co., Ltd., Shanghai, China), 1 mL of enzyme solution was mixed with 1 mL of 0.02 mol L^−1^ phenylalanine, 2 mL of 0.05 mol L^−1^ borate buffer solution (pH 8.8), and the mixture was heated in boiling water for 1 min. The absorbance was measured at 290 nm against reagent blank using an ultraviolet spectrophotometer (Bio-Spectrometer Kinetic, Eppendorf, Hamburg, Germany) after the mixture was placed in a 30 °C water bath for 30 min. The unit of PAL activity was expressed as U, which was defined as the absorbance produced by every 1 g fresh weight sample at 290 nm/h.

### Determination of the PPO and POD activity of *Zizania latifolia*

The POD and PPO activities were determined using Chisari’s method [4] with some modifications. After RF treatments, the samples were tested for PPO and POD activity immediately. The enzymes were extracted by homogenizing 3 g of *Zizania latifolia* in 15 mL of cooled phosphate buffer (pH 7.0, 4 °C) combined with 10 mL of 0.4 mol L^−1^ NaCl solutions. The resulting homogenate was centrifuged at 8000*g* for 10 min at 4 °C (Allegra X-30R Centrifuge). The supernatant combined with precipitation washing solution were adjusted to 25 mL with extraction solution and then stored at 4 °C for later use.

For POD analysis, 5.2 mL of 0.1 mol L^−1^ phosphate buffer (pH 7.0), 0.2 mL of 1% *o*-phenylenediamine-ethanol solution and 0.4 mL of 0.3% hydrogen peroxide solution were mixed, and then 0.2 mL of adjusted enzyme solution was added and mixed evenly. The absorbance of the mixture was measured at 430 nm using an ultraviolet spectrophotometer. The unit of POD activity was expressed as U, which was defined as the absorbance produced from every 3 g fresh weight sample per min at 430 nm.

For PPO analysis, 2 mL of 0.1 mol L^−1^ catechol was mixed with 3.8 mL of 0.1 mol L^−1^ phosphate buffers (pH 6.8). After incubation for 10 min at 37 °C, 0.2 mL of adjusted enzyme solution was added, and then the absorbance was obtained at 410 nm every 30 s for 3 min. The enzyme solution of the control group was boiled for 1 min before mixing. The slope of a linear regression curve of absorbance versus time was used to obtain the enzyme activity. The unit of PPO activity was expressed as U, which was defined as the absorbance produced from every 3 g fresh weight sample per min at 410 nm.

### Determination of H_2_O_2_ and O_2_^−^

Hydrogen peroxide and superoxide were analyzed using the method of Saito with slight modifications [20]. For H_2_O_2_, 3 g of *Zizania latifolia* and 10 mL of acetone were mixed and homogenized at 4 °C in an agate mortar, and then the mixture was centrifuged at 12,000*g* for 12 min. The supernatant (1 mL) was mixed with 0.1 mL of 20% TiCl_4_ concentrated hydrochloric acid solution and 0.2 mL of concentrated aqueous ammonia. Then the mixture was centrifuged at 4000*g* for 10 min. The precipitate was washed 3 times with acetone suspension to reduce pigment interference, and then redissolved in 3 mL of 1 mol L^−1^ H_2_SO_4_. The absorbance of solution was measured at 410 nm, and the unit of H_2_O_2_ content was expressed as Δ, which meant the absorbance produced from every 3 g fresh weight sample at 410 nm.

For O_2_^−^, 3 g of *Zizania latifolia* was mixed with 10 mL of 65 mmol L^−1^ phosphate buffer (pH 7.8), 1 mL 10 mmol L^−1^ hydroxylamine hydrochloride and 1 mL 0.1 M EDTA. The mixture was homogenized in an agate mortar and then centrifuged at 12,000*g* for 12 min. The supernatant (2 mL) was mixed with 2 mL of phosphate buffer (pH 7.8), and the mixture was incubated at 25 °C for 20 min. Two mL of the incubated solution was mixed with 2 mL of 17 mmol L^−1^ *p*-aminobenzenesulfonic acid (12 mol L^−1^ acetic acid solvent) and 2 mL of 7 mmol L^−1^ α-naphthylamine. After reacting at 25 °C for 20 min, the test solution was mixed with the same volume of CCl_4_, and after sufficient shaking, the mixture was gradually divided into two phases. The upper aqueous phase was taken to determine the absorbance at 530 nm. The unit of O_2_^−^ content was expressed as Δ, which was defined as the absorbance produced from every 3 g fresh weight sample at 530 nm.

### Statistical analysis

All analyses were conducted with three replicates. Statistical evaluation and linear regression analyses were performed using Excel 2010 (E Microsoft; Redmond, Washington DC, USA). The significance difference between means were compared using Tukey’s multiple range tests with 5% level (*P* < 0.05).

## Results and discussion

### Effect of RF treatment on the lignin content of Zizania latifolia

**Fig. 1:**
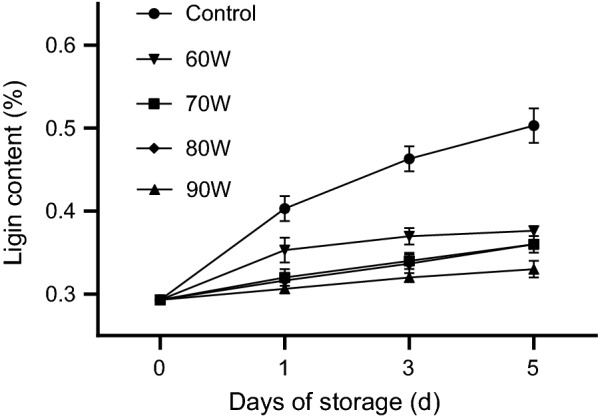
Effect of RF Power on the lignin content of *Zizania latifolia.* Error bars represent the standard errors of the means of four replicates

The lignin content of *Zizania latifolia* is a valued indicator of edible quality. There was as positive correlation (r = 0.96) between hardness and lignin content of *Zizania latifolia* [17]. As shown in Fig. 1, the lignin content of treated groups showed significantly (*P* < 0.05) lower values than that of the control from day 1 to day 5. The result indicated that RF treatments contributed to inhibit the synthesis of lignin during postharvest. Similar results were reported on bamboo shoot with 1-methylcyclopropene treatment [14]. Besides, no significant difference (*P* > 0.05) was observed in groups of 70 W, 80 W and 90 W, indicating that the synthesis of lignin might be largely inhibited when the RF treatment power reached 70 W.

### Effect of RF treatments on the PAL activity of *Zizania latifolia*

**Fig. 2:**
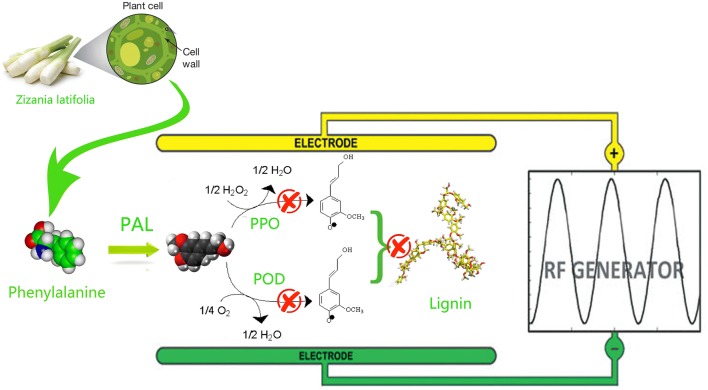
Schematic diagram of RF treatment to inhibit lignin synthesis

**Fig. 3:**
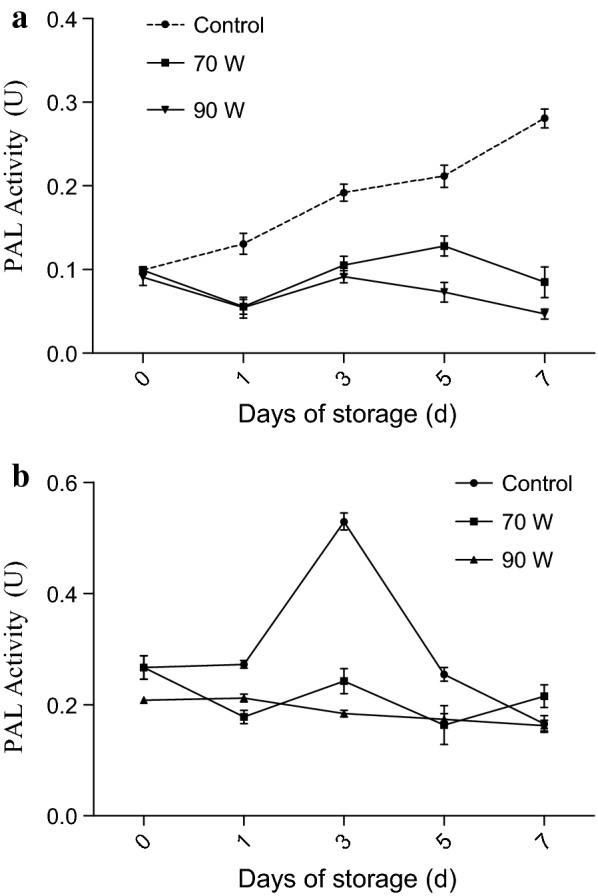
Effect of RF treatments on PAL activity of *Zizania latifolia* (**a** succulent stem; **b** epidermis). Error bars represent the standard errors of the means of three replicates

The lignification of plant tissue is mainly affected by the synthesis of lignin monomer (Fig. 2), while the early study found that the correlation coefficient between PAL enzyme and lignin reached 0.697, suggesting the change of PAL activity could reflect the lignification to some extent [13, 21]. The activity of PAL in succulent stem in the control group increased rapidly during the whole 7 days of storage (Fig. 3a). However, the PAL activities in the RF treatment groups were maintained at a low level. At the 7th day of storage, the PAL activity of the 90 W RF treatment group decreased by 52.9% while the control group increased by 188.5% compared with their initial values. The significant (*P * < 0.05) difference between treated and control groups indicated RF treatments effectively inhibited the PAL activity of succulent stem. However, some fluctuations could be found in Fig. 3a (70 W, 90 W). It may due to incomplete destroy of PAL pathway, and it is possible to synthesize a certain amount of PAL for self-protection. The inhibition effect of RF treatment may be attributed to the following two reasons: first, the thermal effect of RF treatment directly changes the steric configuration of PAL, which makes it difficult to combine with the substrate; second, the high-frequency vibration of the molecule caused by RF treatment are of difficulty in reducing the activation energy of the chemical reaction. The reduction in the deamination efficiency of phenylalanine induced by above reasons resulted in a lack of precursors in the lignification reaction [24]. Besides, there was no significant difference among RF treatment groups on reducing PAL activity (*P* > 0.05). This indicated the possibility of lignification inhibition of Zizania latifolia using smaller RF power.

As shown in Fig. 3b, the initial PAL activity of *Zizania latifolia* epidermis (about 0.2 U) was higher than that of its stem. Previous study shows lignin is mainly deposited in cell walls, providing mechanical support and protection for plants [1, 13]. Therefore, the synthesis rate of lignin and PAL enzyme activity in *Zizania latifolia* epidermis was both higher [5]. In addition, the obvious maximum value of PAL enzyme activity in the control group was observed on 3rd day, which further illustrated that the synthesis rate of lignin in the epidermis was higher than that in stem. The PAL activity was significantly lower than that of the sample without RF treatment, and the inhibition of PAL activity by radio frequency treatment was also demonstrated. For Fig. 3b the upward trend of 70 W on the 3rd day was the same as that of the control group, but the increase was significantly reduced, which proved the inhibition of PAL activity by RF treatment. However, we have not found any research on the difference of enzyme activity between *Zizania latifolia* stem and epidermis for reference. The increase of activity on the 7th day of 70 W group may be due to the self-repair of some plant cells. Several days after RF treatment, a small amount of PAL may be synthesized and led to a renewed increase in enzyme activity. Overall, after RF treatments, the PAL activity in the *Zizania latifolia* epidermis had no significant changes from day 1 to day 7, indicating that RF treatments also inhibited PAL activity in epidermis.

### Effect of RF treatments on POD and PPO activities of *Zizania latifolia*

**Fig. 4:**
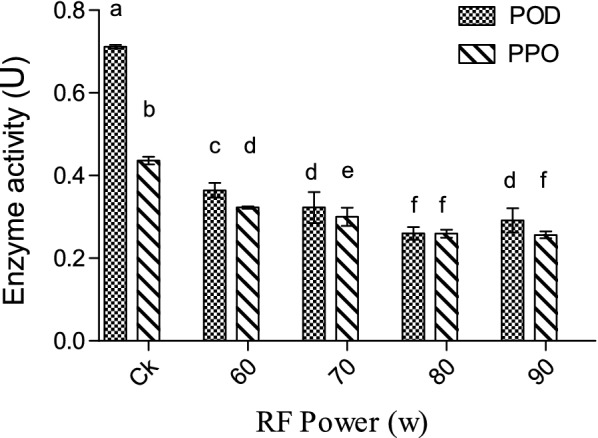
Effect of RF treatments on POD and PPO activities of *Zizania latifolia.* Error bars represent the standard errors of the means of three replicates. Values with different small letters are different at P < 0.05

During lignification process (Fig. 2), PPO is involved in the oxidation of phenolic compounds, catalyzing the formation of caffeic acid as a precursor of lignin synthesized by oxidation of *p*-coumaric acid [6, 22]. Meanwhile, POD can catalyze the decomposition of H_2_O_2_, oxidize and polymerize of lignin monomers, and synthesize lignin macromolecules. From Fig. 4, compared with the control, the activities of POD and PPO in *Zizania latifolia* were significantly (*P * < 0.05) decreased after RF treatment, and the higher inhibition of activities of POD and PPO was observed with the increase of radio frequency power. The activities of POD and PPO decreased by at most 55% and 41% compared to the control, respectively among treated groups. It is widely considered that PPO and POD can form an intermediate complex with the substrate, reducing the energy requirement to break the bond and thereby accelerating the chemical reaction [9]. It is speculated that RF treatment may affect the stability of this intermediate complex or change the partial steric structure of the intermediate composite, resulting in the increased difficulty in catalytic reaction. However, no significant difference (*P* > 0.05) in the inhibition of PPO between 80 and 90 W was observed, indicating that there was no correlation between RF power and activity inhibition of PPO if the power exceeded a certain threshold.

### Effect of RF treatments on the O_2_^−^ and H_2_O_2_ contents of *Zizania latifolia*

**Fig. 5:**
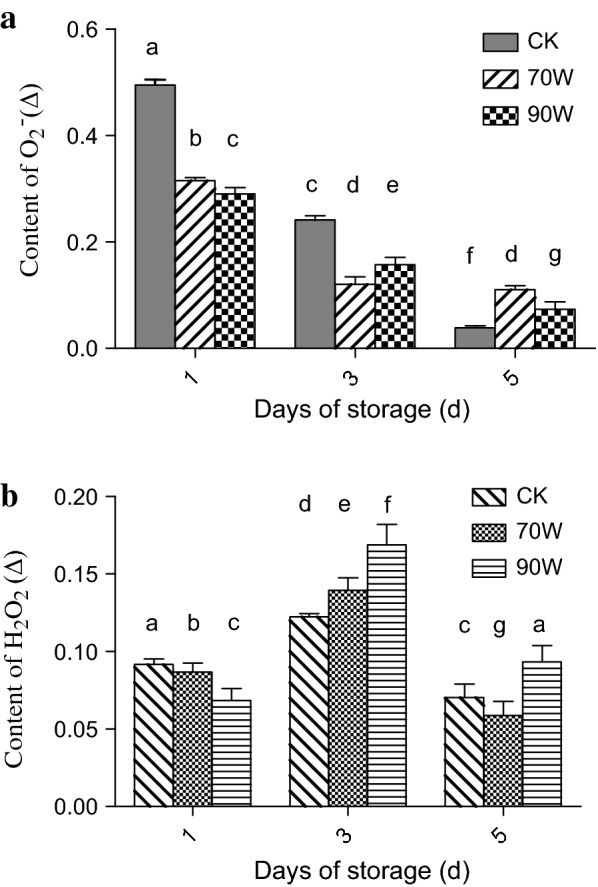
Effect of RF treatments on O_2_^−^ and H_2_O_2_ content of *Zizania latifolia* (**a** O_2_^−^; **b** H_2_O_2_). Error bars represent the standard errors of the means of three replicates. Values with different small letters are different at P < 0.05

Active oxygen is a general term of oxygen single/multi-electron reduction products with extremely strong oxidizing power. The theory of free radicals indicated that the aging of biological tissues is actually the process of metabolic imbalance and accumulation of reactive oxygen species [2]. Under the catalysis of superoxide dismutase (SOD), O_2_^−^ produced in plant tissues can form H_2_O_2_, which plays a crucial role in the cross-linking polymerization of lignin precursors and the lignification of plant tissues combined with POD [25]. Therefore, it is important and beneficial to explore the changes of active oxygen contents after RF treatment. As shown in Fig. 5a, the O_2_^−^ content in the control decreased sharply from day 1 to day 5, and the content on the fifth day was less than 10% of the initial content. It may be due to the rapidly transformation from O_2_^−^ to downstream product of H_2_O_2_ in control samples [20]. Besides, the O_2_^−^ content in the treated groups also decreased rapidly at the beginning and then remained stable, but their final content (day 5) were significantly (*P* < 0.05) higher than that of control group, suggesting that RF treatment contribute to inhibited the activities of enzymes involved in the conversion of O_2_^−^ to downstream products, such as SOD (Eq. 1).

1 $$ 2  {\text{O}}_{2}^{ - } + 2{\text{H}}^{ - } \mathop \to \limits^{SOD} H_{2} O_{2} + O_{2} $$

According to Fig. 5b, the content of H_2_O_2_ in *Zizania latifolia* in all groups showed the similar change trends, with the initial increase and subsequent decline. The contents of H_2_O_2_ in the treated groups were significantly (*P* < 0.05) higher than that in the control group on day 3, which was consistent with the result of O_2_^−^ (Fig. 5a) because O_2_^−^ was rapidly converted to H_2_O_2_ under enzymatic catalysis. More residual H_2_O_2_ in *Zizania latifolia* indicated that less H_2_O_2_ participated in the lignification process [3]. Besides, it also indirectly suggested that related enzymes such as POD were inhibited since the lignification process required the assistance of these enzymes.

## Conclusions

The results in this study confirmed that RF treatments effectively inhibited the synthesis of lignin of *Zizania latifolia* in storage, related enzyme activities and active oxygen evaluations. The lower lignin content and the activities of PAL, POD and PPO were observed in *Zizania latifolia* treated with RF treatment when compared to the control. Besides, RF treatment also decreased the conversion of O_2_^−^ to H_2_O_2_ by inhibiting the related enzyme activities. The results suggested that RF treatment had the great potential to delay lignification of *Zizania latifolia*. Future investigation is required to optimize the conditions of RF treatment to obtain better inhibition effect of lignification and to consider this technology to *Zizania latifolia* processing industry. In addition, the impact of RF treatment on the quality of *Zizania latifolia* also needs to be considered.

## Data Availability

The datasets used and/or analyzed during the current study are available from the corresponding author on reasonable request.

## Abbreviations

RF: radio frequency
PAL: phenylalanine ammonia lyase
POD: peroxidase
PPO: polyphenol oxidase
CAD: acetaldehyde dehydrogenase
O_2_^−^: superoxide anion
H_2_O_2_: hydrogen peroxide
PVP: polyvinylpyrrolidone
SOD: superoxide dismutase
